# Supplementary material for: Thin-Film Engineering of Mechanical Fragmentation Properties of Atomic-Layer-Deposited Metal Oxides
Source: Nanomaterials (Basel). 2020 Mar 19;10(3):558. doi: 10.3390/nano10030558 (PMC7153380; doi:10.3390/nano10030558)
Supplement: Supplementary file 1 [file nanomaterials-10-00558-s001.pdf]

Supplementary Information

Thin-Film Engineering of Mechanical Fragmentation Properties of Atomic-Layer-Deposited Metal Oxides

Mikko Ruoho [1], Janne-Petteri Niemelä [1], Carlos Guerra-Nunez [1], Natalia Tarasiuk [1], Georgina Robertson [1], Aidan A. Taylor [2], Xavier Maeder [1], Czeslaw Kapusta [3], Johann Michler [1], and Ivo Utke [1,*]

1 Empa–Swiss Federal Laboratories for Materials Science and Technology, Laboratory for Mechanics of Materials and Nanostructures, Feuerwerkerstrasse 39, CH-3602 Thun, Switzerland; mruoho@iki.fi (M.R.); janne-petteri.niemelae@empa.ch (J.N.); Carlos.Guerra-Nunez@empa.ch (C.G.); tarasiuknatalia@gmail.com (N.T.); georginar018@gmail.com (G.R.); xavier.maeder@empa.ch (X.M.); Johann.Michler@empa.ch (J.M.)

2 Materials Department, University of California, Santa Barbara, 93106 California, USA; aidantaylor@ucsb.edu

3 AGH University of Science and Technology Krakow, Faculty of Physics and Applied Computer Science, Al.Mickiewicza 30, 30-059 Kraków, Poland; kapusta@agh.edu.pl

* Correspondence: ivo.utke@empa.ch; Tel.: +41-58-765-6257

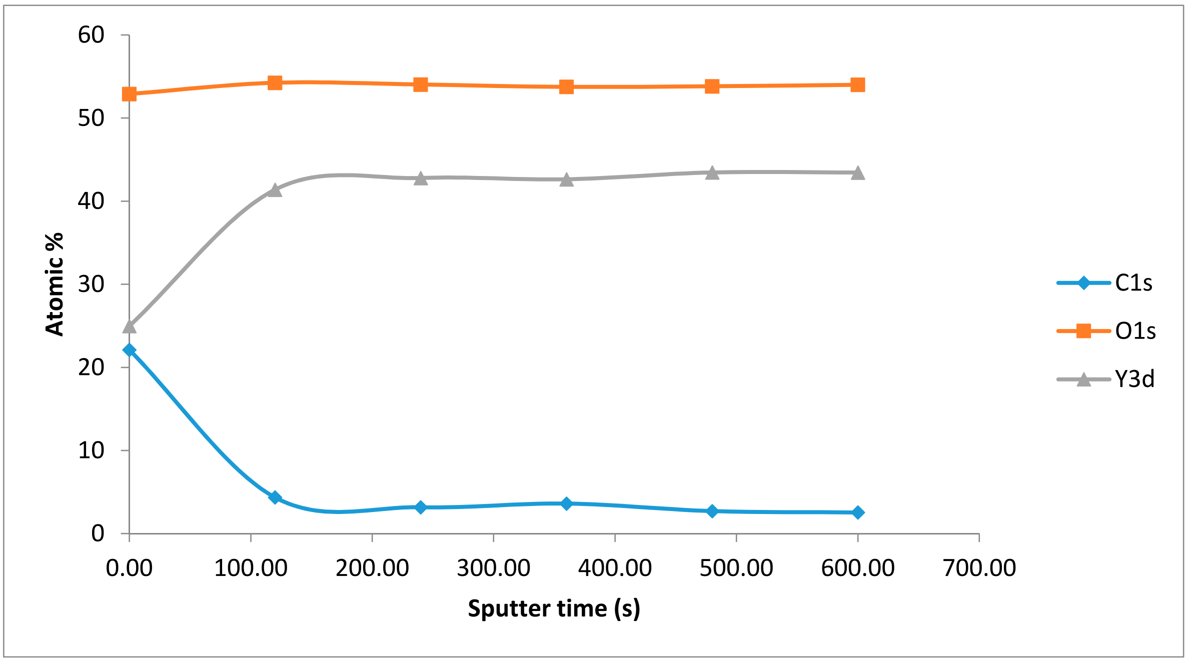

Figure S1. XPS depth profile of Y_2O_3 sample.

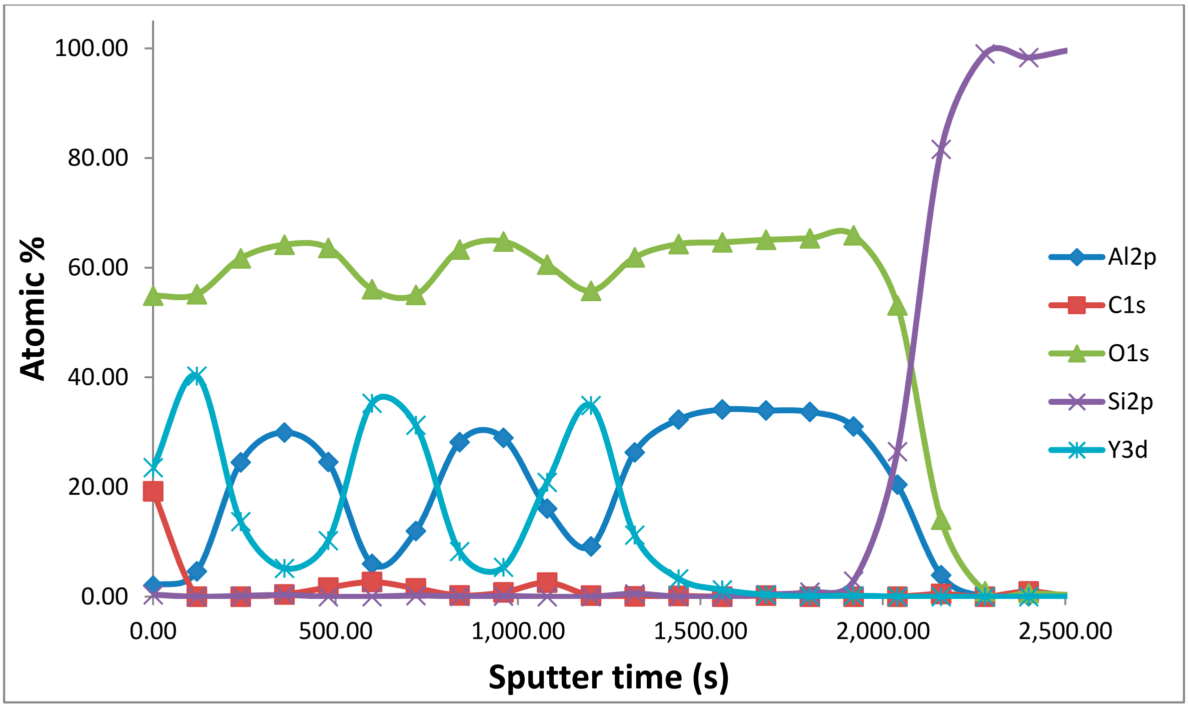

Figure S2. XPS depth profile of 3 × NL (Y_2O_3–Al_2O_3) on Al_2O_3 sample.
